# A Milk-Fat Based Diet Increases Metastasis in the MMTV-PyMT Mouse Model of Breast Cancer

**DOI:** 10.3390/nu13072431

**Published:** 2021-07-15

**Authors:** Fabiola N. Velazquez, Valentina Viscardi, Julia Montemage, Leiqing Zhang, Carolena Trocchia, Megan M. Delamont, Rasheed Ahmad, Yusuf A. Hannun, Lina M. Obeid, Ashley J. Snider

**Affiliations:** 1 Department of Medicine, Stony Brook University, Stony Brook, NY 11794, USA; FabiolaNoelia.Velazquez@stonybrookmedicine.edu (F.N.V.); ValentinaViscardi7@gmail.com (V.V.); julia.montemage@gmail.com (J.M.); leiqing.zhang.1@stonybrookmedicine.edu (L.Z.); carolena.trocchia@gmail.com (C.T.); yusuf.hannun@stonybrookmedicine.edu (Y.A.H.); lina.obeid@stonybrookmedicine.edu (L.M.O.); 2 Cancer Center, Stony Brook University, Stony Brook, NY 11794, USA; 3 Department of Nutritional Sciences, College of Agriculture and Life Sciences, University of Arizona, Tucson, AZ 85721, USA; delamontmegan@gmail.com; 4 Immunology & Microbiology Department, Dasman Diabetes Institute, Kuwait City 15462, Kuwait; rasheed.ahmad@dasmaninstitute.org

**Keywords:** diet, fatty acid, breast cancer, lung metastasis

## Abstract

A high-fat diet (HFD) and obesity are risk factors for many diseases including breast cancer. This is particularly important with close to 40% of the current adult population being overweight or obese. Previous studies have implicated that Mediterranean diets (MDs) partially protect against breast cancer. However, to date, the links between diet and breast cancer progression are not well defined. Therefore, to begin to define and assess this, we used an isocaloric control diet (CD) and two HFDs enriched with either olive oil (OOBD, high in oleate, and unsaturated fatty acid in MDs) or a milk fat-based diet (MFBD, high in palmitate and myristate, saturated fatty acids in Western diets) in a mammary polyomavirus middle T antigen mouse model (MMTV-PyMT) of breast cancer. Our data demonstrate that neither MFBD or OOBD altered the growth of primary tumors in the MMTV-PyMT mice. The examination of lung metastases revealed that OOBD mice exhibited fewer surface nodules and smaller metastases when compared to MFBD and CD mice. These data suggest that different fatty acids found in different sources of HFDs may alter breast cancer metastasis.

## 1. Introduction

Obesity is as an independent risk factor for the development of breast cancer [1]. The consumption of dietary fat increases the risk of developing breast cancer [2,3,4] with several studies linking increased breast cancer incidence to a high-fat Western diet [5,6,7,8]. Furthermore, obesity has also been associated with increased tumor size and poor prognosis [9,10]. However, the contribution of specific fat sources and fatty acids in those sources (saturated fatty acid (SFA), monounsaturated fatty acid (MUFA), and polyunsaturated fatty acid (PUFA)) to increased breast cancer risk is less clear [11]. Two meta-analysis studies identified associations between the increased risk of breast cancer and a higher intake of SFA [12,13], while data from a matched control study in Sweden determined that there was a positive correlation with MUFA and PUFA intake, but not SFA intake [14]. Other reports have shown that MUFAs and PUFAs could have independent and opposite effects on breast cancer, with MUFA inversely and PUFA positively associated with breast cancer risk [15,16]. Additionally, the intake of specific types of PUFA have been shown to have an inverse association with the risk of breast cancer [17,18]. Several factors can contribute to these contradictory results, such as errors in measuring dietary fat, studies in populations with uniform fat intake, or the fact that specific types of dietary fat are present in the same food sources. The use of mouse models to evaluate the effects of diet on breast cancer development allows us a more controlled environment to study the effect of different fat sources.

The mammary polyomavirus middle T antigen overexpression mouse model (MMTV-PyMT) is a powerful tool to examine the effects of diet in the development of breast cancer. Mammary lesions in these mice closely resemble human breast cancer progression [19] and respond to dietary modification [20]. Previous reports have shown that a high-fat diet (HFD) from soybean oil increased primary mammary tumors as well as lung metastasis in MMTV-PyMT mice. The up-regulation of proinflammatory cytokines, adipokines, and angiogenic factors were associated with the increased aggressiveness of mammary carcinogenesis caused by HFD [20,21]. Deletion of the adipocyte MCP-1 gene in MMTV-PyMT mice decreased the levels of pro-inflammatory cytokines and angiogenic factors caused by HFD feeding and attenuated its effect on tumor growth [22], denoting the close connection between obesity–inflammation–breast cancer in this mouse model. An additional benefit to examining the effects of specific types of fat in a HFD in the MMTV-PyMT mice on the FVB background is that this background strain is resistant to diet-induced obesity [23,24]. This allows for the study of dietary effects on tumorigenesis and metastasis without the confounding effects of obesity.

The present study tested the effects of two different HFDs on primary mammary tumor formation and pulmonary metastasis in the MMTV-PyMT mouse model of breast cancer. A milk fat-based diet (MFBD) and an olive oil-based diet (OOBD) were compared to an isocaloric low-fat control diet (CD) (Table 1). No differences were detected in tumor latency or growth among the different diets, while the MFBD but not OOBD, increased lung metastasis compared to the CD. These results suggest that different sources of dietary fat and potentially specific dietary fatty acids may influence breast cancer progression in the MMTV-PyMT model.

## 2. Materials and Methods

### 2.1. Mouse Models

MMTV-PyMT (FVB/N-Tg (MMTV-PyMT) 634 Mul/J) mice were purchased from The Jackson Laboratory. MMTV-PyMT female mice were used for this study. Mice were maintained on regular chow until 8 weeks of age, at which time they were randomized to either a milk fat-based diet (MFBD, 42% of calories from fat, TD.088137), an olive oil-based diet (OOBD, 42% of calories from fat, TD.140589), or an isocaloric control diet (CD, 12.6% of calories from fat, TD.05230) purchased from Envigo, Inc. (Indianapolis, IN, USA). Animals were maintained under standard laboratory conditions, and all animal procedures were approved by the Institutional Animal Care and Use Committee at Stony Brook University (SBU) and followed the guidelines of the American Veterinary Medical Association.

### 2.2. Tumor Monitoring

Tumor monitoring and measurements were performed as described in Velazquez et al. [25]. The area of the lung metastases were calculated through microscopy (EVOS M500, Thermo Fisher Scientific, Waltham, MA, USA).

### 2.3. RNA Extraction and Quantitative Real-Time PCR

RNA extraction and quantitative real-time PCR in tumor tissues were performed as described in Choi et al. [26]. The following TaqMan probes (Thermo Fisher Scientific, Waltham, MA, USA) were used: mouse S1PR1 (ID: Mm02619656_s1), mouse TNFα (ID: Mm00443260_g1), mouse MMP9 (ID: Mm00442991_m1), and mouse β-actin (ID: Mm02619580_g1). Cycle threshold (Ct) values were obtained for each gene of interest and normalized to the β-actin.

### 2.4. Statistical Analysis

Statistical analyses were performed using GraphPad Prism (GraphPad Software, San Diego, CA, USA). Data are mean ± S.E.M. and were analyzed by one-way ANOVA with a Dunn’s Multiple Comparison Test or by an unpaired Student’s *t*-test. *p* < 0.05 was considered statistically significant.

## 3. Results

### 3.1. Milk Fat and Olive Oil Diets Do Not Alter Tumor Weight or Volume in MMTV-PyMT Mice

**Figure 1:**
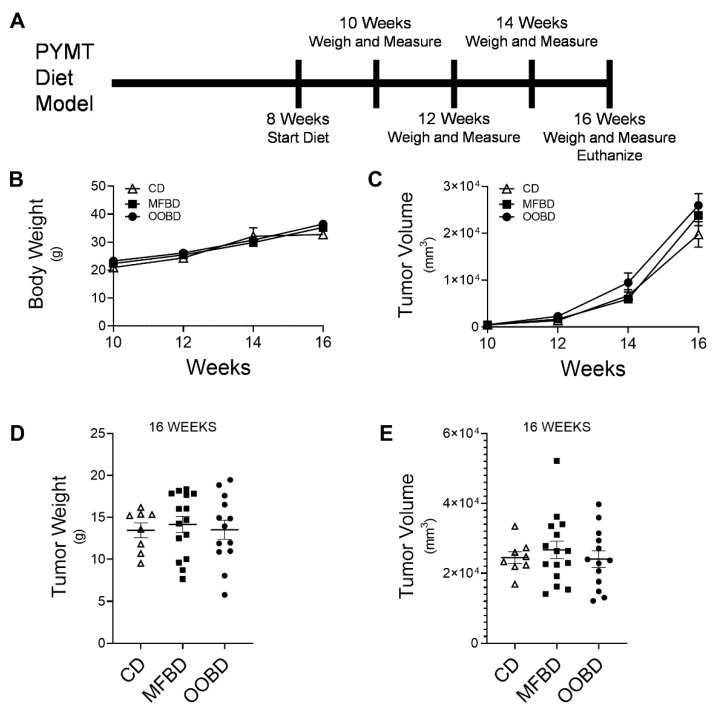
High fat diets (HFDs) do not alter tumor volume or weight in the mammary polyomavirus middle T antigen MMTV-PyMT breast cancer model. (**A**) Scheme for diets and MMTV-PyMT breast cancer model. Female MMTV-PyMT mice on the FVB background were fed a milk fat-based diet (MFBD), olive oil-based diet (OOBD), or an isocaloric control diet (CD) beginning at 8 weeks of age weeks. (**B**) Body weight was assessed every two weeks. (**C**) Tumor volume was measured at 10, 12, 14, and 16 weeks of age using calipers. (**D**,**E**) Tumors were excised at 16 weeks and weighed, and tumor volume was measured with calipers. Data represent mean ± standard error of the mean (SEM), n ≥ 8 for each diet.

Obesity and diets high in fat have been shown to have a significant impact on the prognosis and survival in breast cancer patients [27,28]. To begin to define the impact of diets enriched with specific dietary fatty acids on breast cancer, we used female MMTV-PyMT mice, which present with high tumor incidence and metastases rates [29,30]. These mice provide more rapid and predictable metastases that develop in the lung and lymph nodes. Female MMTV-PyMT mice were fed a MFBD, OOBD, or CD starting at 8 weeks of age. Body weight and tumor measurements were collected every two weeks, and the mice were euthanized at 16 weeks (Figure 1A). From 10 to 16 weeks, the body weights were similar among all diets including the CD (Figure 1B). Mice fed MFBD and OOBD exhibited no difference in tumor weight or final tumor volume from CD fed mice at 16 weeks (Figure 1C–E). Importantly, CD fed mice exhibited similar tumor growth to MMTV-PyMT mice who were fed a standard regular chow diet (data not shown). These data suggest that a MFBD nor an OOBD alter primary tumor size or tumor weight in the MMTV-PyMT model.

### 3.2. MFBD Fed Mice Exhibit Increased Lung Metastasis in MMTV-PyMT Mice

**Figure 2:**
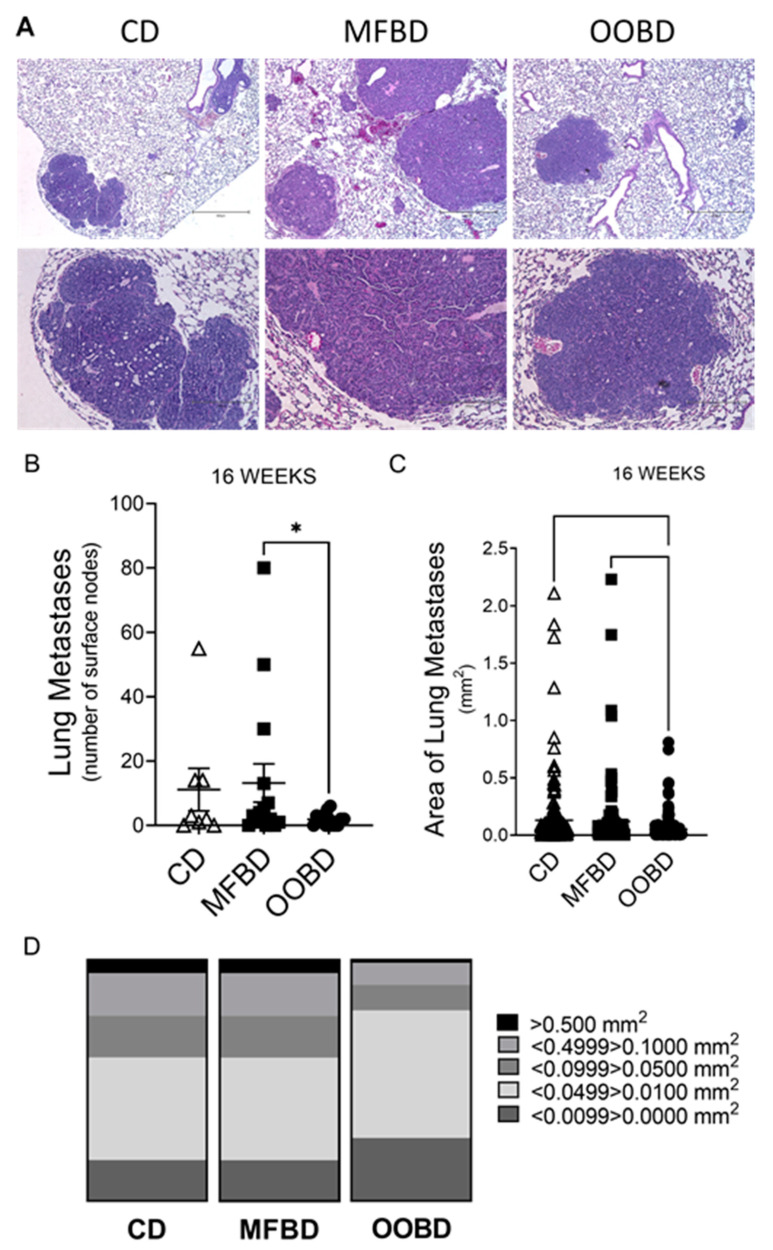
MFBD increases lung metastases in the MMTV-PyMT breast cancer model. (**A**) Representative images of hematoxylin and eosin (H&E) stained lung sections from MMTV-PyMT mice fed CD, MFBD, or OOBD. Upper panel magnification = 4x, scale bar = 600 µm; lower panel magnification = 10x, scale bar = 250 µm. (**B**) Quantification of the number of surface nodes on the lungs from 16-week-old MMTV-PyMT fed CD, MFBD or OOBD. Data represent mean ± SEM. Statistical analysis: Unpaired *t*-test, * *p* < 0.05, n ≥ 8 for each diet. (**C**) Quantification of the area of lung metastasis mice fed CD, MFBD, or OOBD. Data represent mean ± SEM. Statistical analysis: one-way ANOVA with a Dunn’s multiple comparison test, * *p* < 0.05, n = 4 for each diet. (**D**) Tumor size distribution represented as percent of total.

HFDs have been implicated in metastasis and inflammation in MMTV-PyMT mice [31]. Therefore, in order to determine if the MFBD or OOBD altered lung metastases in the MMTV-PyMT mice, lungs were harvested at 16 weeks and assessed for metastases (Figure 2A). The number of surface nodules were significantly higher in mice who were fed the MFBD (13.20 ± 5.96, mean ± SEM) than the mice who were fed the OOBD (1.93 ± 0.55) (Figure 2B, CD = 11.43 ± 6.60). Moreover, the area for the lung metastases from OOBD (average: 0.52 × 10^5^ µm^2^ ± 0.83 × 10^4^) was significantly smaller when compared to the CD (average: 1.30 × 10^5^ µm^2^ ± 2.34 × 10^4^) or the MFBD (average: 1.21 × 10^5^ µm^2^ ± 2.79 × 10^4^) mice (Figure 2C). Large tumors (>0.1 mm^2^) were present in 23% of the CD and MFD mice, but in only 10% of the OOBD mice, indicating mice fed the OOBD exhibited smaller metastases (Figure 2D). These results indicate that while the MFBD does not increase primary tumor growth, this diet results in increased lung metastasis in the MMTV-PyMT model.

### 3.3. MFBD Increases TNFα Expression in Primary Tumors

**Figure 3:**
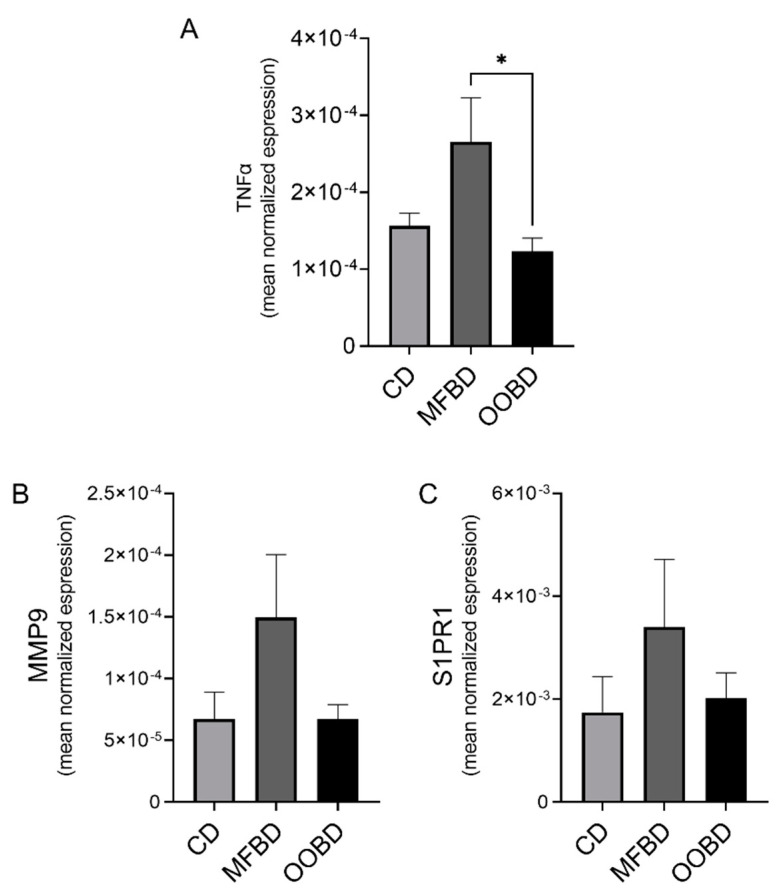
MFBD increases tumor necrosis factor alpha (TNFα) expression in primary tumors in the MMTV-PyMT breast cancer model. mRNA levels of (**A**) TNFα, (**B**) matrix metalloproteinase 9 (MMP9), and (**C**) sphingosine-1-phosphate receptor 1 (S1PR1) expression in primary tumors were analyzed using real time RT-PCR and normalized to β-actin. Data represent mean ± SEM. Statistical analysis: Unpaired *t*-test, * *p* < 0.05, n ≥ 6 for each diet.

Many factors influence primary tumor growth and overall metastatic potential including VEGF, HIF1α, and FGF as well as many cytokines and chemokines. Therefore, we next investigated the effects of enriched HFDs on the signaling pathways and inflammation in the tumor. Using a RT-PCR array, we surveyed 42 growth factors, cytokines, and chemokines that have been implicated in tumor growth, inflammation, and metastases (Table S1). From the array, several MMPs, cytokines, and sphingosine-1-phosphate receptor 1 (S1PR1) were of particular interest. Upon validation, primary tumor tissues from the MFBD mice exhibited significant increases in TNFα expression compared to tumors from the OOBD fed mice as well as increased an expression of S1PR1 and MMP9; however, these were not statistically significant (Figure 3). These cytokines suggested the potential for a more inflammatory tumor micro-environment in mice fed a MFBD than an OOBD.

## 4. Discussion

The present study demonstrates that two different types of HFDs do not affect tumor latency or primary tumor growth in the MMTV-PyMT model compared to an isocaloric control diet. However, mice fed an OOBD exhibited decreases in the number of lung metastases (assessed by surface nodules) compared mice fed a MFBD. Additionally, OOBD fed mice exhibited a decreased area of lung metastasis compared to the CD and MFBD mice. These results suggest that different types of HFDs could have a differential effect on breast cancer progression without affecting primary tumor growth.

The absence of differences in tumor latency and primary tumor growth contradicts previous studies in the MMTV-PyMT model where a HFD prolonged [21] or shortened [32] tumor latency and increased tumor growth [20,21,32]. One of the reasons for these observed discrepancies could be the age when the diet was started in the female mice. MMTV-PyMT mice are a fast-progressing model with mammary lesions starting as early 4 weeks of age and signs of early malignant transition at 8 weeks of age [30]. The present work analyzed tumor progression in mice fed either a CD, a MFBD, or an OOBD from 8–16 weeks of age, while the previous reports that show an effect on primary tumor growth started at 3–4 weeks of age [20,21,32]. Additionally, we did not observe an increase in weight gain in female mice fed with either MFBD or OOBD compared to the CD. This is somewhat not surprising as the FVB background strain has been shown to be resistant to diet induced obesity [23,24]. In a study by Hu et al., FVB mice fed a HFD for 8 weeks did not exhibit changes in body weight, metabolic rate, or the expression of nutrient-sensitive genes [24]. In fact, ovariectomized FVB mice fed with HFD at 8 weeks of age for 13 weeks and injected with MMTV-PyMT tumor derived cells did not show differences in tumor growth as well as in body weight [33]. These results suggest that in the lack of obesity, a high-fat diet alone does not modify primary tumor growth.

The higher levels of TNFα expression in the primary tumor tissues from MFBD mice compared to OOBD and CD mice could play role in the differences observed in metastasis. The increase of TNFα and additional cytokines have been previously reported in mammary tumors from MMTV-PyMT mice fed with a HFD [21]. TNFα has been shown to induce epithelial–mesenchymal transition in breast cancer cell lines [34,35], and it has been suggested as a prognostic marker for human breast cancer progression [36,37]. Interleukin-6 (IL6) has also been implicated in mammary tumors from mice fed a HFD [38] and in tumor cell lines derived from MMTV-K-Ras mice [39]. Due to the significance of the local immune environment in breast cancer progression [40], with findings that also show its role in the MMTV-PyMT model [41], additional studies on the tumor microenvironment and inflammatory cytokines could help to elucidate mechanisms contributing to the observed differences in metastases.

Additional factors from the diet used in the present study could also play a role in the results observed. For example, it has been shown that HFD caused hyperinsulinemia when compared to a control diet [42]. Insulin could affect cell growth and alter the invasiveness of breast cancer cells [43], and insulin resistance has been shown to be associated with increased breast cancer risk [44]. The MFBD and OOBD used in this work have similar calories from carbohydrates (43.7% Kcal and 43% Kcal, respectively) and their percentages are lower than in the CD (68.5% Kcal). Plasma insulin levels or an additional low carbohydrate diet could lend insight into the role of insulin and insulin resistance in tumor formation and metastasis in HFD models.

Studies over the last 20 years have associated a Mediterranean diet (MD), which is typically associated with consumption of olive oil, with lower breast cancer incidence in humans across several different study populations [45,46,47,48]. When compared to a Western diet (WD) it has been suggested that the MD could be preventive in the development of breast cancer [49]. However, a study examining the effects of a MD in France demonstrated no association between breast cancer risk and MD [50]. The PREDIMED study uncovered associations of decreased breast cancer risk in women that consumed a MD with extra virgin olive oil when compared to a MD with nuts or an advised low-fat control diet [51]. Our studies found no difference in overall tumor growth or progression among the MFBD, the OOBD, or CD. Future studies should be geared at examining additional sources of dietary fatty acids on initial tumor growth and progression, as this could lend important insight into sources of dietary fats to examine in future clinical studies.

Analysis of lung metastasis in mice fed the OOBD demonstrated a significant reduction in the average area of lung metastasis compared to the CD and MFBD, while comparison between the two HFDs indicated that the OOBD decreased the number of surface nodes compared to the MFBD. Previous reports have shown increased primary tumor growth and metastases in MMTV-PyMT mice fed either a soybean oil based HFD [21,22] or a lard based HFD [30] compared to a control or standard chow diet. Conversely, a study by Cowen et al. demonstrated higher tumor volume but not increased lung metastases in MMTV-PyMT mice fed a lard based HFD compared to a low-calorie control diet [20]. These studies demonstrate the importance of an isocaloric control diet and the use of two or more fat sources for investigations into the effects of a specific HFD on breast cancer development and metastasis. Our study is the first study reporting a positive effect of a specific type of HFD, OOBD vs. MFBD, on breast cancer progression in MMTV-PyMT mice by decreasing the area of lung metastasis. Future studies will be geared at defining the mechanisms by which specific dietary fatty acids in HFDs alter metastasis and breast cancer development.

## Figures and Tables

**Table 1:** Diet Composition.

Diet Formula	Control		MFBD		OOBD	
	(g/Kg)		(g/Kg)		(g/Kg)	
Casein	195.00		195.00		195.00	
DL-Methionine	3.00		3.00		3.00	
Sucrose	341.00		341.46		345.20	
Corn Starch	211.99		150.00		147.82	
Maltodextrin	100.00		-		-	
Anhydrous Milkfat	37.20		210.00		72.50	
Olive Oil	-		-		135.50	
Soybean Oil	12.80		-		-	
Cholesterol	-		1.50		1.85	
Cellulose	50.00		50.00		50.00	
Mineral Mix, AIN-76	35.00		35.00		35.00	
Vitamin Mix, Teklad	10.00		10.00		10.00	
Ethioxyquin, antioxidant	0.01		0.04		0.04	
** Nutrient Composition **	**% Weight**	**% Kcal**	**% Weight**	**% Kcal**	**% Weight**	**% Kcal**
Protein	17.30	18.70	17.30	15.20	17.30	15.30
Carbohydrate	63.50	68.60	48.50	42.70	48.70	43.00
Fat	5.20	12.60	21.10	42.00	21.00	41.70
Energy (Kcal/g)	3.70		4.50		4.50	
** Diet Formula **	**g/Kg**	**%**	**g/Kg**	**%**	**g/Kg**	**%**
Total Fat	58.98		216.55		223.64	
SFA	26.10	52.4	136.71	65.2	70.40	33.85
MUFA	14.60	29.3	65.73	31.3	120.70	70.40
PUFA	9.10	18.3	7.35	3.5	16.90	13.95
4:0	1.40	2.37	7.98	3.69	2.76	1.23
6:0	0.86	1.46	4.83	2.23	1.67	0.75
8:0	0.41	0.70	2.31	1.07	0.80	0.36
10:0	0.74	1.25	4.20	1.94	1.45	0.65
12:0	1.20	2.03	6.51	3.01	2.25	1.01
14:0	4.40	7.46	24.57	11.35	8.48	3.79
14:1	0.30	0.51	1.68	0.78	0.58	0.26
15:0	0.60	1.02	3.36	1.55	1.16	0.52
16:0	11.20	18.99	55.02	25.41	37.60	16.81
16:1	0.71	1.20	3.99	1.84	3.00	1.34
17:0	0.26	0.44	1.47	0.68	0.51	0.23
17:1	-	0.00	0.04	0.02	0.15	0.07
18:0	5.20	8.82	26.25	12.12	12.50	5.59
18:1	13.50	22.89	59.22	27.35	116.80	52.23
18:2	7.90	13.39	6.09	2.81	15.70	7.02
18:3	1.20	2.03	1.05	0.48	1.18	0.53
20:1	-	0.00	0.42	0.19	0.15	0.07
20:4	-	0.00	0.21	0.10	0.00	0.00
*n-6*	1.20	2.03	6.30	2.91	15.70	7.02
*n-3*	7.90	13.39	1.05	0.48	1.20	0.54

MFBD = milk fat-based diet; OOBD = olive oil-based diet; g = gram; Kg = kilogram; AIN = American Institute of Nutrition; Kcal = kilocalorie; SFA = saturated fatty acid; MUFA = monounsaturated fatty acid; PUFA = polyunsaturated fatty acid; *n-6* = omega-6; *n-3* = omega-3.

## Supplementary Materials

The following is available online at https://www.mdpi.com/article/10.3390/nu13072431/s1, Table S1: PCR Array analysis.
