# Supplementary material for: A Milk-Fat Based Diet Increases Metastasis in the MMTV-PyMT Mouse Model of Breast Cancer
Source: Nutrients. 2021 Jul 15;13(7):2431. doi: 10.3390/nu13072431 (PMC8308868; doi:10.3390/nu13072431)
Supplement: Supplementary file 1 [file nutrients-13-02431-s001.zip › nutrients-1263720-supplementary.pdf]

Gene	**Expression**<br>(Fold change over CD)	
	MFBD	OOBD
Angpt1	1.70	0.71
Angpt2	1.10	0.73
Bmp4	2.09	1.75
Brms1	0.85	0.56
Ccl2	0.62	1.43
Ccl20	1.11	1.10
Ccl5	6.10	3.38
Cdh1	1.51	0.72
Cerk	0.90	0.61
Ctnnb1	1.73	0.87
Cxcl1	1.27	0.84
Cxcl2	1.50	0.69
Egf	1.09	0.50
Dnajb9	1.13	2.84
Fgf1	3.65	1.44
Fgf2	3.59	1.23
Hif1a	1.09	0.79
Il6	0.58	0.52
Cxcl15	0.41	0.08
Mmp2	0.71	0.36
Mmp9	1.40	1.94
Mmp14	1.74	1.45
Myc	1.08	0.51
Nme1	0.76	0.60
Pdgfa	1.69	1.07
Pecam1	1.12	0.66
Ptgs2	2.60	2.00
S1pr1	2.20	6.50
Sphk1	0.28	0.39
Smad4	1.32	0.72
Spns2	2.18	0.78
Tgfb1	0.89	0.74
Tgfb2	0.60	0.32
Tgfb3	1.10	0.55
Timp1	1.88	1.00
Timp2	1.69	0.72
Tnf	3.61	2.92
Vegfa	1.49	0.72
Vegfb	1.12	1.14
Vegfc	6.13	5.63
Wnt1	1.09	1.56
Wnt5a	1.92	0.79
